# A Rare Multi-vessel Spontaneous Coronary Artery Dissection Case Requiring Balloon Intervention

**DOI:** 10.7759/cureus.55164

**Published:** 2024-02-28

**Authors:** Abdelgani A Abdelgani, Rafaqat Hussain, Hayfa O Ahmed, Stephen Thomson, Waleed Mohamed

**Affiliations:** 1 Cardiology, Scarborough General Hospital, Scarborough, GBR; 2 Internal Medicine, Scarborough General Hospital, Scarborough, GBR

**Keywords:** multi-vessel scad required intervention, a rare scad case required intervention, spontaneous coronary artery dissection requiring intervention, multivessel scad, atypical spontaneous coronary artery dissection

## Abstract

Spontaneous coronary artery dissection (SCAD) is a rare cause of acute coronary syndrome. This case involves a multivessel SCAD requiring intervention. The patient is a 39-year-old woman suffering from a non-ST elevation myocardial infarction caused by SCAD. The first coronary angiography revealed changes suggestive of acute distal left anterior descending (LAD) spontaneous dissection with partial occlusion and changes suggestive of old distal left anterior circumflex artery and obtuse marginal spontaneous dissections. A repeated angiogram revealed total occlusion of the distal LAD. Balloon angioplasty was done to the distal LAD, achieving a good flow. This case highlights the importance of diagnosis and treatment of SCAD. This case enhances our knowledge of atypical SCAD presentation (multi-vessel and required intervention) and emphasizes the need for individualized management strategies for optimal outcomes in each case.

## Introduction

Spontaneous coronary artery dissection (SCAD) is an uncommon and potentially life-threatening disease involving disruption of the innermost layer of a coronary artery. This tear leads to blood coagulation between the arterial walls and eventually results in a blood clot. If this clot blocks even just part of the lumen, this may cause a heart attack [1].

This health condition mostly affects women, especially those who are young and otherwise healthy [2,3]. The exact cause of SCAD is not well understood, and it can occur in individuals without typical risk factors for heart disease. However, some factors that may be associated with SCAD include hormonal changes, such as those occurring during pregnancy or postpartum [4], connective tissue disorders, such as fibromuscular dysplasia or Ehlers-Danlos syndrome, and certain genetic factors [5].

Clinical manifestations such as chest pains or discomfort, difficulty breathing, and general feeling of tiredness in SCAD are like those found in other types of acute coronary syndrome. The patient can also present with atypical symptoms [6]. Diagnosing SCAD can be challenging. Imaging techniques such as coronary angiography or intravascular ultrasound may be used to detect the tear in the artery. However, these tears can be subtle and may not always be easily identified [7].

The management of SCAD depends on the severity of the condition. However, in some instances, conservative management may suffice, but in other serious cases, stenting and bypass operations might still be necessary. Treatment is approached individually according to the condition of the patient [8]. Outcomes in individuals with SCAD vary. Some patients will regain their normal health with medical assistance, while some can have repeated symptoms. Long-term follow-up and treatment are critical. Lifestyle modifications may also be required to reduce recurrence events [8].

As SCAD is a complicated and somewhat uncommon disorder, people who suffer from it should be treated by specialized healthcare teams proficient in managing this kind of disease. There are studies going on currently toward understanding its predisposition and the best treatment modalities for SCAD. We describe a 39-year-old female patient with SCAD causing acute myocardial infarction highlighting the diagnostic and management challenges.

## Case presentation

The patient, a 39-year-old female with a medical history notable for migraine, hypertension, and cholecystectomy, initially experienced chest pain that resolved days before admission. However, recurrent chest pain radiating to the back and left arm resulted in her hospitalization. Although the electrocardiogram (ECG) on admission showed no concerning abnormalities, elevated troponin levels (785 ng/L) and negative findings on aortic imaging warranted further investigation.

On the initial presentation, the intensity scale of her chest pain was 4/10 in severity; therefore, we decided to proceed with a coronary artery angiogram. An echocardiogram was done at the emergency department revealed akinesis of the apical anteroseptal and apical anterior segments, indicating a potential cardiac ischemic event.

**Figure 1:**
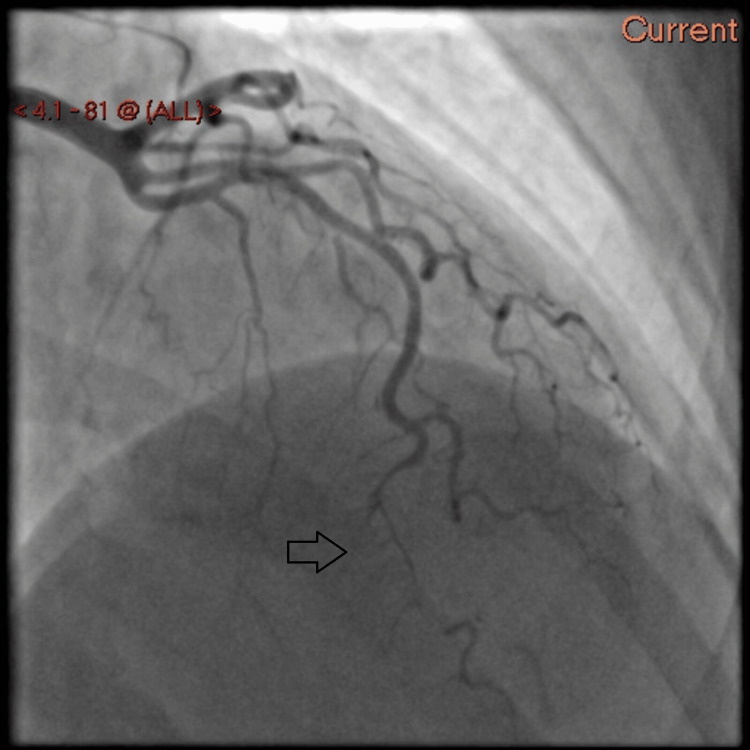
Long segment dissection in the mid-distal LAD LAD: Left anterior descending

Subsequent coronary angiograms unveiled a long segment dissection in the mid-distal LAD, as shown in Figure 1, and involvement of the left anterior circumflex artery/obtuse marginal (OM).

**Figure 2:**
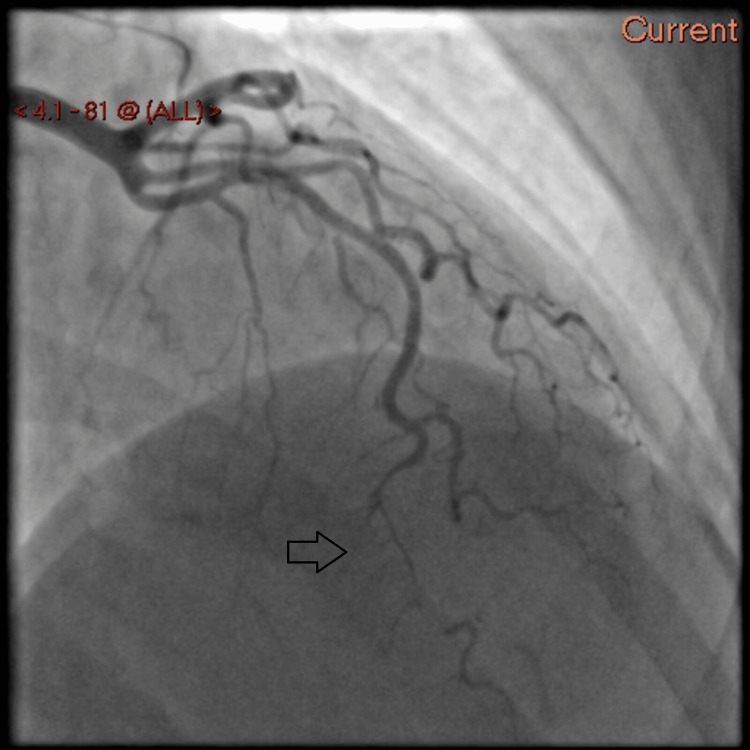
Repeated coronary angiogram showing further impairment of the flow in the distal LAD LAD: Left anterior descending

The decision for conservative management was initially made, but due to ongoing chest pain, a repeat angiogram was done, which revealed occlusion in the distal LAD shown in Figure 2. At that stage, the decision was made to intervene in the distal LAD.

**Figure 3:**
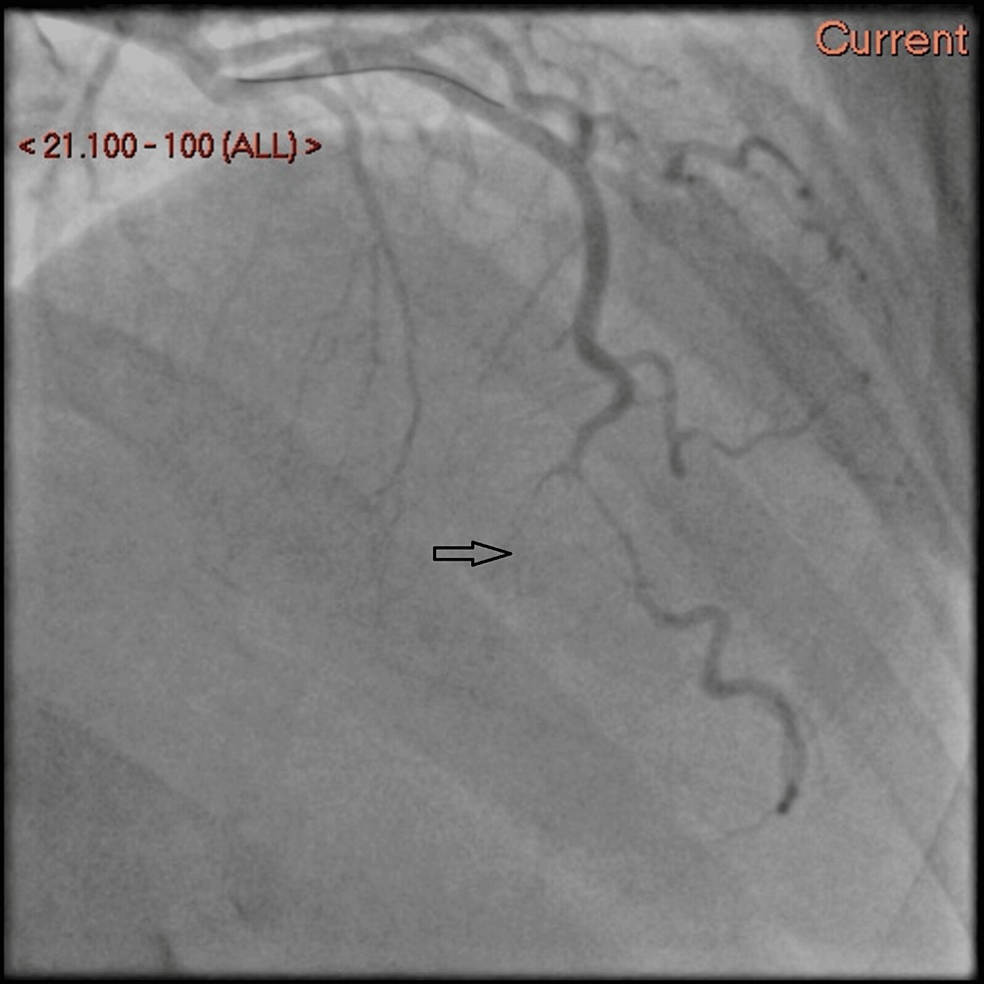
Restored flow to distal LAD post-intervention with percutaneous balloon angioplasty LAD: Left anterior descending

A timely decision was made to proceed with percutaneous balloon angioplasty to the distal LAD. The procedure resulted in a favorable outcome, restoring the flow as shown in Figure 3. Remarkably, an additional dissection involving the OM was discovered, potentially representing a previous event unrelated to the acute presentation.

The patient was initiated on dual antiplatelet therapy (aspirin 75mg once daily lifelong; ticagrelor for one year), secondary prevention medications (bisoprolol, ramipril, atorvastatin), and nitro-glycerine. Post-angioplasty, the patient experienced transient T-wave inversion in V4, V5, V6, II, III and AVF.

During follow-up, the patient demonstrated resolution of symptoms and improved left ventricular ejection fraction (LVEF), estimated at 55%. The patient was maintained on a comprehensive medical regimen for secondary prevention.

## Discussion

Despite numerous studies conducted on SCAD, it still persists as a challenging diagnostic and therapeutic problem, especially for non-ST elevation myocardial infarction patients in young, low-risk populations [9]. This case highlights several noteworthy aspects contributing to the evolving understanding of SCAD. Our patient’s unusual clinical presentation consisting of a repeated episode of chest pain after resolution of the initial interval highlights the importance of having a high index of suspicion for SCAD, particularly when dealing with non-traditional risk markers for SCAD. In addition, no significant ECG findings were present at admission, showing that SCAD is challenging to detect initially [10]. An angiogram initially depicted a distally impaired flow in LAD, which presents difficulties in diagnostics on SCAD. This dynamic nature of SCAD necessitates constant monitoring and reassessment, as shown in the progressive flow reduction to warrant immediate intervention.

The use of a multi-disciplinary team approach is a valuable strategy for managing SCAD (Namkoong et al., 2023) [11]. In this case, despite initial conservative management recommendations, the persistence of symptoms and a subsequent decline in coronary flow prompted a shift toward intervention. This decision highlights the necessity for individualized patient care and underscores the limitations of generalizable treatment algorithms for SCAD.

The decision to perform balloon angioplasty in the setting of ongoing symptoms and deteriorating flow in the distal LAD represents a departure from the typical conservative approach advocated for SCAD. This case raises questions about the appropriate timing and indications for intervention in SCAD, emphasizing the need for further research to guide evidence-based therapeutic strategies.

While intriguing, the discovery of an additional dissection involving the OM artery adds complexity to the case. The challenge lies in differentiating acute from chronic dissections and establishing a causal relationship with the presenting symptoms [12]. This aspect underscores the need for comprehensive angiographic assessment and the potential for uncovering additional complexities in SCAD cases.

Post-intervention, the patient demonstrated symptomatic improvement and an increase in LVEF. The selection of dual antiplatelet therapy and secondary prevention medications aligns with current guidelines, emphasizing the importance of long-term care and close follow-up in SCAD patients.

As our understanding of SCAD continues to evolve, this case underscores the critical need for further research. Prospective studies exploring optimal diagnostic strategies, refining indications for intervention, and elucidating the long-term implications of multiple dissections are essential. A collaborative, multidisciplinary approach is fundamental to advancing the knowledge base and improving outcomes in SCAD.

## Conclusions

This case adds a significant contribution to the growing understanding of SCAD. It highlights the need for accurate diagnosis, continuous evaluation, and personalized treatment approaches. Although conservative management is often preferred for many SCAD cases, there are times when intervention becomes necessary. Enhancing long-term care for this rare and challenging condition necessitates further research.
